# Molecular Determinants of Species Specificity of α-Conotoxin TxIB towards Rat and Human α6/α3β4 Nicotinic Acetylcholine Receptors

**DOI:** 10.3390/ijms24108618

**Published:** 2023-05-11

**Authors:** Ting Xie, Yuan Qin, Jinyuan Zhao, Jianying Dong, Panpan Qi, Panpan Zhang, Dongting Zhangsun, Xiaopeng Zhu, Jinpeng Yu, Sulan Luo

**Affiliations:** 1 School of Medicine, Guangxi University, Nanning 530004, China; 2028391024@st.gxu.edu.cn (T.X.);; 2 Key Laboratory of Tropical Biological Resources, Ministry of Education, Key Laboratory for Marine Drugs of Haikou, Hainan University, Haikou 570228, China

**Keywords:** α-conotoxin TxIB, α6/α3β4 nicotinic acetylcholine receptor, species specificity, electrophysiology

## Abstract

Conotoxins are widely distributed and important for studying ligand-gated ion channels. TxIB, a conotoxin consisting of 16 amino acids derived from *Conus textile*, is a unique selective ligand that blocks rat α6/α3β2β3 nAChR (IC_50_ = 28 nM) without affecting other rat subtypes. However, when the activity of TxIB against human nAChRs was examined, it was unexpectedly found that TxIB had a significant blocking effect on not only human α6/α3β2β3 nAChR but also human α6/α3β4 nAChR, with an IC_50_ of 537 nM. To investigate the molecular mechanism of this species specificity and to establish a theoretical basis for drug development studies of TxIB and its analogs, different amino acid residues between human and rat α6/α3 and β4 nAChR subunits were identified. Each residue of the human species was then substituted with the corresponding residue of the rat species via PCR-directed mutagenesis. The potencies of TxIB towards the native α6/α3β4 nAChRs and their mutants were evaluated through electrophysiological experiments. The results showed that the IC_50_ of TxIB against h[α6_V32L, K61R_/α3]β4_L107V, V115I_ was 22.5 μM, a 42-fold decrease in potency compared to the native hα6/α3β4 nAChR. Val-32 and Lys-61 in the human α6/α3 subunit and Leu-107 and Val-115 in the human β4 subunit, together, were found to determine the species differences in the α6/α3β4 nAChR. These results also demonstrate that the effects of species differences between humans and rats should be fully considered when evaluating the efficacy of drug candidates targeting nAChRs in rodent models.

## 1. Introduction

Nicotinic acetylcholine receptors (nAChRs) are a group of pentameric ligand-gated ion channels that are sensitive to nicotine. nAChRs in mammals can be classified as muscle-type nAChRs consisting of α1, β1, and γ or ε, δ, and various neural-type nAChRs comprising α2–α7, α9, α10, and β2–β4 subunits which, today, are potential drug targets, mainly in the case of neural-type acetylcholine receptors [1,2,3]. The physiological functions of natural nAChRs containing α6 subunits are complex, and there are limited studies on their function and expression distributions, which are broadly divided into two isoforms: α6β2* (asterisks indicate possible additional subunits in the natural receptors), constructed of β2 subunits, and α6β4*, comprising β4 subunits. The α6* nAChRs in the central nervous system (CNS) are predominantly α6β2* subtypes distributed in several limited and discrete brain regions. They are understood to be associated with the regulation of dopamine release in reward and addiction [4,5,6,7]. The α6β2* nAChRs are potential therapeutic targets for the treatment of neuropsychiatric disorders, including addiction and Parkinson’s disease [8,9,10].

In contrast, little is currently known about the role of α6β4* nAChRs in the peripheral nervous system. The α6β4* nAChRs have restricted distribution in rat dorsal root ganglia (DRGs) [11,12]. As the primary neuron for nociceptive afferents, the dorsal root ganglion transmits and regulates the proprioception, reception, and transmission of injurious sensations, playing an essential role in the pain mechanism. The ion channels and their receptors, being closely related to the pain mechanism, are key to achieving targeted analgesia in the DRG. Recent studies have found that α6β4* nAChRs expressed in the DRG interact directly with and cross-inhibit P2X2/3 receptors. Strains with high levels of CHRNA6 expression show lower levels of mechanical nociception in several neuropathic and inflammatory pain models, resulting in neuropathic pain symptoms that are inversely correlated with the level of CHRNA6* expression [1,13]. The study of the essential functions of α6β4* nAChRs is beneficial for the development of novel neuropathic pain drugs [11]. As previously reported for the α6 subunit, attempts to express the rat and human nAChR α6 subunit with β4 and β2 (α6β4 and α6β2 nAChRs) in *Xenopus* oocytes consistently failed; that is, no ACh-gated currents could be detected. To improve the functional expression, we used rα6/α3 and hα6/α3 (chimeric nAChRs of α6 and α3 isoforms) instead of the corresponding wild-type α6 [14,15]. The mutants used in this study are all combinations of human α6/α3β4 and rat α6/α3β4 N-terminal extracellular structural domain (ECD) differential site mutations, and the α6/α3β4 species always match one another.

The pathogenesis of neuropathic pain is commonly explored using rodent models. It is also used in screening research on several potential drugs modulating pain transmission and perception. However, rodent models may be somewhat compromised due to differences in ligand sensitivity between receptors and ion channels in humans and rodents. It has been reported that the reduced sensitivity of nAChRs to Vc1.1 in humans, relative to rats, may have contributed to the poorer analgesic effect of Vc1.1 in human clinical trials [16]. Therefore, it is important to determine how species differences affect receptor–ligand interactions [1]. The search for highly selective and potent blockers of α6β4* nAChRs is significant for studying their physiology and fundamental functions. Therefore, the search for critical amino acid sites of α6β4* nAChRs affecting drug sensitivity is important and can provide a molecular basis for the search for other nAChR isoforms that can be distinguished with similar structures and overlapping distributions, such as α3β4 and α6β2* nAChRs [17,18].

TxIB, a conotoxin consisting of 16 amino acids found in *Conus textiles*, specifically blocks rα6/α3β2β3 nAChR, with an IC_50_ value of 28 nM. It has no effect on rα6/α3β4 nAChR (IC_50_ > 10,000 nM) and is one of the best ligands available that interact with α6/α3β2β3 nAChR [19]. In contrast, when examining the action of TxIB on human nAChRs, it was found to block hα6/α3β4, with an IC_50_ value of 537 nM [20,21]. In this study, we constructed single-amino-acid substitution mutations. We evaluated the activity of receptor mutants with TxIB, aiming to investigate the molecular mechanisms responsible for this species specificity and provide a pharmacological basis for the development of TxIB-related pharmacological tools. This research also provides a molecular basis for functional and fundamental studies of α6/α3β4 nAChR and the development of α6*-nAChR-targeted medications and neuropathic pain drug leads targeting α6β4 nAChRs [22].

## 2. Results

### 2.1. Synthesis and Identification of TxIB

**Figure 1:**
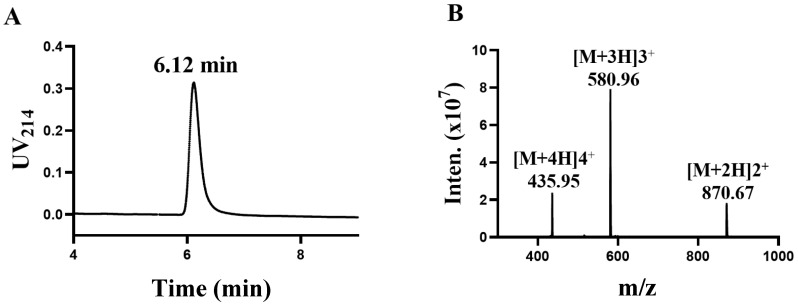
Synthesis and identification of α-conotoxin TxIB (**A**,**B**). (**A**) The purified peptide TxIB was analyzed with an analytical RP-HPLC. (**B**) Electrospray ionization mass spectrometry (ESI-MS) data with an observed mass of 1739.70 Da.

TxIB is an α conotoxin containing 16 amino acid residues with the sequence GCCSDPPCRNKHPDLC# (# C-terminal carboxamide). In a previous study, intrinsic differences between human and rat α6/α3β4 nAChRs conferred different sensitivities to α-CTxs [20]. The determination was performed using high-performance liquid chromatography (HPLC). The central peak was collected, and the results are shown in Figure 1A, showing that the central absorption peak appeared at 6.12 min. Afterwards, the exact relative molecular mass of α-conotoxin TxIB was identified using mass spectrometry (Figure 1B). The relative molecular mass of α-conotoxin TxIB was calculated to be 1739.88 Da via mass spectrometry, which is consistent with the theoretical value of 1739.99 Da.

### 2.2. Identification of Crucial Residues Affecting the Blocking Activity of Human β4 Subunits with TxIB

**Figure 2:**
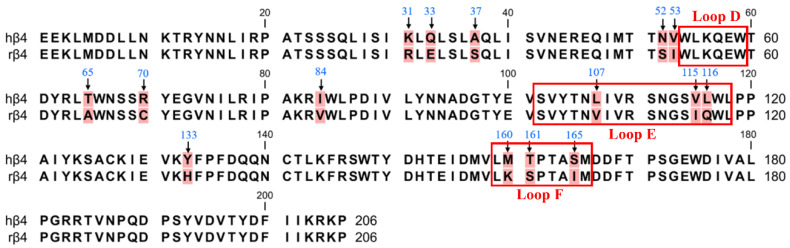
The amino acid sequence alignment of the extracellular N-terminal domain of hβ4 and rβ4 subunits. There are 15 different sites (background marked in red). The positions that were mutated in this study are indicated with arrows. The boxes refer to the region of residues in the β4 subunit that form the ligand-binding pocket.

Previous reports have shown that in a pentameric nAChR with three β subunits and two α subunits, the site of ligand binding is the interface formed by the α and β subunits, and thus, it is widely believed that the β4 subunit is the major subunit for ligand binding in α6/α3β4 nAChR [1,23,24]. Therefore, we first focused on the role of the β4 subunit in contributing to species differences via PCR-mediated mutagenesis. The ligand-binding pocket is a hydrophobic pocket-like region consisting of a series of relatively conserved aromatic amino acids located at the interface of the α-subunit (complementary subunit) and the β-subunit (primary subunit) in nAChRs. The α6/α3 subunits form the ligand-binding pocket consisting of loops A (residues 81–91), B (residues 144–149), and C (residues 187–194) [25]. The β4 subunits form the ligand-binding pocket consisting of loops D (residues 54–59), E (residues 102–118), and F (residues 159–166) [26]. Many studies have revealed that the amino acid residues in the ligand-binding pocket play a vital role in ligand binding and channel function [27,28,29]. Sequence analysis of the β4 subunit revealed a total of 15 differential sites, with the most widely observed sequence differences occurring at positions 107, 115, and 116, which are Leu, Val, and Leu in human β4 and Val, Ile, and Gln in rats (Figure 2). These three nonconserved residues are all located in the ligand-binding pocket loop E and may be critical amino acid sites that are responsible for the α6/α3β4 species differences [27]. Among the human β4 differential sites, Asn-52 and Val-53 are close to loop D, while Met-160, Thr-161, and Ser-165 are located in loop F. Loops D, E, and F are ligand-binding regions of the ligand β4 subunit and are associated with ligand binding [30,31].

Mutations in the receptor may cause functional changes. Therefore, to assess the receptor function of α6/α3β4 nAChR mutants, EC_50_ values were determined for the mutants in the presence of different concentrations of ACh. The resulting EC_50_ values are shown in Table 1, and most mutants showed no significant changes in function. hα6/α3β4_N52S_ could not form a functional receptor and might be related to the expression of the receptor. The only mutation site that showed potential substantial changes was the S165I mutation in β4, whose EC_50_ value of 50 μM was 2.6-fold lower than that of 138 μM for the human α6/α3β4 nAChR (Table 1). A37S, V53I, L107V, and M160K had slightly reduced EC_50_ values compared to hα6/α3β4 nAChR (Table 1). The EC_50_ values of the K31R, R70C, I84V, V115I, and L116Q mutants of the β4 subunit changed very little, being almost indistinguishable from hα6/α3β4 nAChR (Table 1). The remaining four single-point mutants of the β4 subunit (Q33E, T65A, Y133H, T161S) had slightly larger EC_50_ values compared to hα6/α3β4 (Table 1).

**Figure 3:**
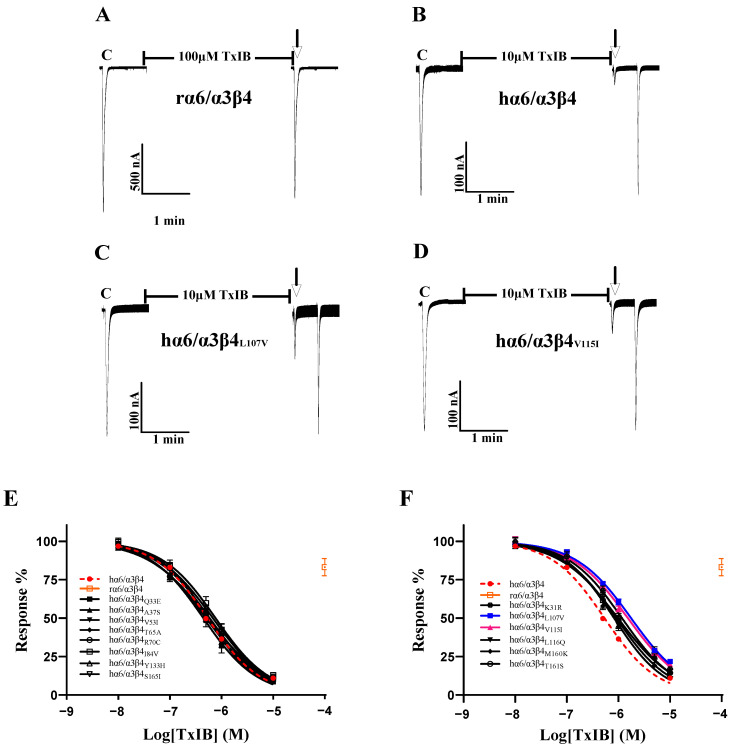
The activity of TxIB towards α6/α3β4 and its β4 mutant nAChRs (**A**–**F**). (**A**–**D**) Representative responses of the WT α6/α3β4 and mutant nAChRs are shown. “C” indicates the control responses to ACh without TxIB incubation. The arrows indicate the current generated upon ACh stimulation after 5 min of TxIB incubation and washing to enable the recovery of the blockade. The specific method is described in the electrophysiological assay section. Representative 100 μM ACh-evoked currents obtained in the presence of 100 μM TxIB for the human and rat WT α6/α3β4 nAChRs (**A**,**B**). Results of 10 μM TxIB for the mutant α6/α3β4_L107V_ (**C**) and α6/α3β4_V115I_ (**D**). (**E**,**F**) Concentration–response curves for TxIB inhabitation of hα6/α3β4 nAChRs and all β4 subunit mutants. β4 mutants with similar activity to WT (**E**). β4 mutants with reduced activity compared to WT (**F**). IC_50_ values and Hill slopes are given in Table 2.

As the sequence analysis of the β4 subunit revealed six amino acid sites that are not conserved in residues that constitute the ligand-binding pocket, particularly Leu-107, Val-115, and Leu-116, which were all found to be critical in previous studies, Leu-116 is the most critical amino acid site for the species specificity of α-conotoxin PeIA for human and rat α6/α3β4 [1]. TxIB acts with significant species specificity on human and rat α6/α3β4 nAChRs; 100 μM TxIB had almost no blocking effect on rα6/α3β4, and 10 μM TxIB completely blocked hα6/α3β4 (Figure 3A,B). The inhibition values of the current response to 100 μM ACh were 21.73 ± 4.29% (n = 10) and 20.1 ± 3.86% (n = 8) for the L107V and V115I mutants, respectively, following incubation with 10 μM TxIB for 5 min, compared with 10.99 ± 5.9% (n = 16) for hα6/α3β4 under the same conditions. The IC_50_ values of TxIB were 1760 nM for the L107V mutant and 1525 nM for the V115I mutant, with 3.3-fold and 2.8-fold decreases in potency, respectively, compared to WT hα6/α3β4 (Table 2, Figure 3A–D). In particular, the blocking effect of TxIB on the 116-site mutation changed very little compared to hα6/α3β4, with the IC_50_ becoming only 1.7-fold larger, whereas it was previously reported that the IC_50_ value of PeIA acting on hα6/α3β4_L116Q_ versus the WT hα6/α3β4 was nearly 10-fold greater [1]. Concerning residues located in the ligand-binding pocket region, hα6/α3β4_S165I_ had almost identical blocking effects compared to human α6/α3β4, while hα6/α3β4_M160K_ and hα6/α3β4_T161S_ showed slight changes in blocking, increasing 1.5-fold and 1.8-fold, respectively (Table 2). Usually, we would consider a change in IC_50_ of 3-fold or more as a significant difference. We performed electrophysiological experiments on all the remaining β4 subunit single-point mutations based on the sequence comparison results to determine whether there were more prominent residues. The blocking effect of the hα6/α3β4_K31R_ mutant was also slightly altered, with a 2.2-fold increase in the IC_50_ value compared to hα6/α3β4 (Figure 3F). The remaining Q33E, A37S, V53I, T65A, R70C, I84V, and Y133H mutants varied within a small range and were insignificant (Figure 3E).

### 2.3. Identification of Crucial Residues Affecting the Blocking Activity of Human α6/α3 Subunits with TxIB

**Figure 4:**
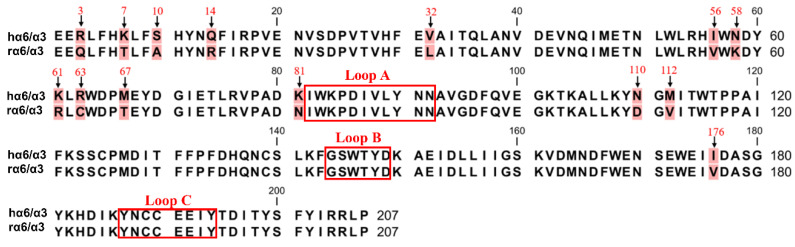
The amino acid sequence alignment of the extracellular N-terminal domain of hα6/α3 and rα6/α3 subunits. There are 14 different sites (background marked in red). The positions that were mutated in this study are indicated with arrows. The boxes refer to the region of residues in the α6/α3 subunit that form the ligand-binding pocket.

The sequence comparison between the human and rat α6/α3 subunit N-terminus extracellular structural domain (ECD) showed 14 amino acid residue differential sites, and none of them were located in the ligand-binding region (Figure 4). We mutated the amino acid sites in the ECD of the α6/α3 subunit, and among the 14 differential sites, Asn-58, Lys-61, Lys-81, Asn-110, and Met-112 were associated with the expression of α6* nAChRs [25]. The site that was spatially closest to the ligand-binding pocket was Ile-176, and the remaining eight differential sites were reported to be almost irrelevant [1,6].

The EC_50_ values of the R3Q, K7T, V32L, I56V, N58K, R63C, M67T, M112V, and I176V mutants in the α6/α3 subunit did not change significantly under the influence of ACh compared to the natural hα6/α3β4 nAChR (Table 3). The S10A, Q14R, K61R, K81N, and N110D mutations in the α6/α3 subunit showed a slight increase in their EC_50_ values compared to hα6/α3β4 nAChR (Table 3). Among all the single-point mutations in the α6/α3 subunit, the K61R mutation had the largest change in its EC_50_ value of 202 μM, which is a 1.5-fold increase compared to the value of hα6/α3β4 nAChR.

**Figure 5:**
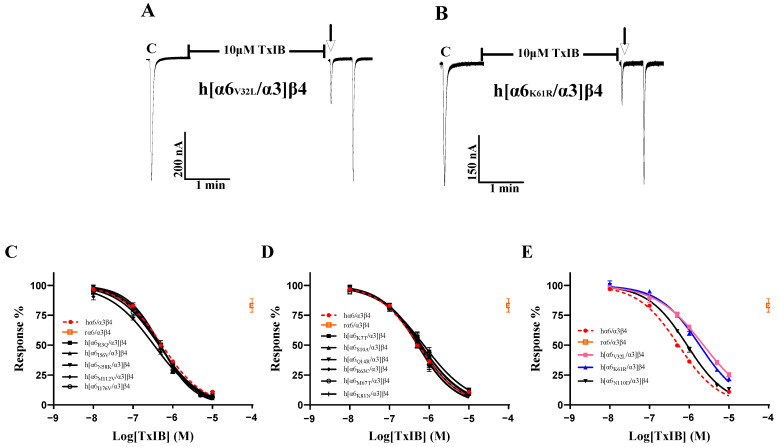
The activity of TxIB towards hα6/α3β4 and its α6/α3 mutant nAChRs (**A**–**E**). “C” indicates the control responses to ACh without TxIB incubation. The arrows indicate the current generated upon ACh stimulation after 5 min of TxIB incubation and washing to enable the recovery of the blockade. Representative ACh-evoked currents were obtained in the presence of 10 μM TxIB for h[α6_V32L_/α3]β4 (**A**) and h[α6_K61R_/α3]β4 (**B**). Concentration–response curves for TxIB inhabitation of hα6/α3β4 nAChRs and all α6/α3 mutants. Activity enhancement compared to WT (**C**); α6/α3 mutants with similar activity to hα6/α3β4 (**D**); α6/α3 mutants with reduced activity compared to hα6/α3β4 (**E**). IC_50_ values and Hill slopes are given in Table 4.

To investigate the key residues in α6/α3 that interact with TxIB, we first focused on five differential loci associated with the functional expression of α6* nAChRs. The results showed a significant decrease in the blocking effect of TxIB after hα6/α3 underwent K61R mutation, with a 3.4-fold-increased IC_50_ of 1.83 μM (Table 4). The inhibition of the current response of the K61R mutant to 100 μM ACh following incubation with 10 μM TxIB was 22.07 ± 4.7% (n = 8) (Figure 5B). The blocking results of the N58K, K81N, N110D, and M112V mutations interacting with TxIB were similar, with slightly enhanced blocking effects of N58K and M112V, with IC_50_ values of 471 nM and 337 nM, respectively, and slightly reduced blocking effects of K81N and N110D, with IC_50_ values of 634 nM and 884 nM, respectively (Table 4, Figure 5C–E). Hone reported that the isoleucine at position 176 is close to the ligand-binding structural domain [1]. Therefore, we were interested in assessing whether the sensitivity of h[α6_I176V_/α3]β4 to TxIB differs from that of the wild-type hα6/α3β4 subtype, and the results showed almost no difference, with an IC_50_ of 451 nM being very similar to the IC_50_ value of hα6/α3β4 (Table 4). For a further comprehensive study, we tested the activity of all the single-point mutant receptors to determine whether there were more critical sites on the α6/α3 subunit. Among the remaining eight differential sites, h[α6_V32L_/α3]β4, with a large difference in activity, showed a noticeable change in the IC_50_ value and a 4.2-fold decrease in the blocking effect (Table 4). The inhibition of the current response of the h[α6_V32L_/α3]β4 mutant to 100 μM ACh following incubation with 10 μM TxIB was 25.3 ± 5.23% (n = 7) (Figure 5A). In summary, the α6 subunit also plays an important role in species differences, with the key amino acid residues being Val-32 and Lys-61.

### 2.4. Identification of Multiple Amino Acid Residues of TxIB That Are Highly Effective against Human α6/α3β4 nAChR

**Figure 6:**
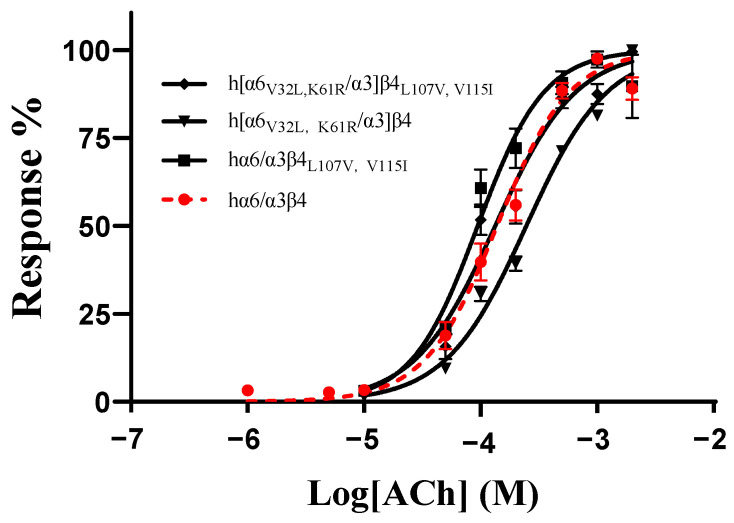
Concentration–response curves of acetylcholine (ACh) activation for the WT and all combined mutants of hα6/α3β4 nAChRs. EC_50_ values and Hill slopes are given in Table 5.

As no mutants with significant changes in activity were found in all the single-point mutations in the ECD (N-terminal extracellular structural domain) of the human and rat α6/α3 and β4 subunits, we hypothesized that the residues affecting the differences in the action of blocking the activity of TxIB are multilocus interactions. According to the results above, the mutants with the greatest changes in activity in the β4 subunit were L107V and V115I, and the mutants with the greatest changes in activity in the α6/α3 subunit were V32L and K61R. To assess the effect of comutation on the action of ACh on the mutants, we determined the response rates of the mutants at different ACh concentrations. The results showed that hα6/α3β4_L107V, V115I_ had an EC_50_ value of 94 μM, with only a slight change, while h[α6_V32L, K61R_/α3]β4 had a relatively significant change in its EC_50_ value of 246 μM, with a 1.8-fold increase. In contrast, the EC_50_ of the mutant h[α6_V32L, K61R_/α3]β4_L107V, V115I_ was 113 μM, which is almost indistinguishable from that of the wild-type hα6/α3β4 (Table 5, Figure 6).

**Figure 7:**
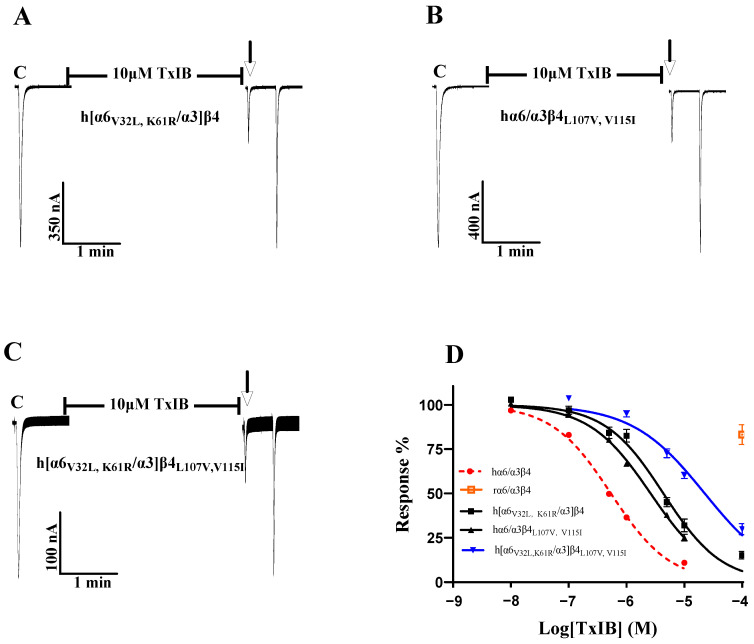
Activity of TxIB towards the WT and mutant α6/α3β4 nAChRs (**A**–**D**). “C” indicates the control responses to ACh without TxIB incubation. The arrows indicate the current generated upon ACh stimulation after 5 min of TxIB incubation and washing to enable the recovery of the blockade. Representative ACh-evoked currents obtained in the presence of 10 μM TxIB for hα6/α3β4_L107V, V115I_ (**A**), h[α6_V32L, K61R_/α3]β4 (**B**), and h[α6_V32L, K61R_/α3]β4_L107V, V115I_ (**C**). Concentration–response curves for TxIB inhabitation of hα6/α3β4 nAChRs and their combined mutants (**D**). IC_50_ values and Hill slopes are given in Table 6.

As the single-point mutations failed to identify key sites, we considered the comutagenesis of amino acid sites that exhibited significant changes in single-point mutations in the β4 subunit and α6/α3 subunit and explored multiple sites affecting species differences in TxIB. To investigate the changes in the interaction of multiresidue mutations in the β4 subunit with TxIB, hα6/α3β4_L107V_ and hα6/α3β4V115I (the two sites with the greatest changes in blocking activity in the β4 subunit single-point mutations) were prepared. Table 5 shows that the IC_50_ value of hα6/α3β4_L107V,V115I_ acting on TxIB was 2.6 μM, which was 4.9-fold greater than that of hα6/α3β4, and the blocking activity was further reduced compared with L107V (a 3.3-fold greater IC_50_ value compared with hα6/α3β4) and V115I (a 2.8-fold greater IC_50_ value compared with hα6/α3β4). Oocytes expressing the hα6/α3β4_L107V, V115I_ mutant showed 25.11 ± 11.21% inhibition (n = 8) of the current response to 100 μM ACh after 5 min of incubation at 10 μM TxIB (Figure 7B). Along the same lines as the β4 subunit, h[α6_V32L, K61R_/α3]β4 (encompassing the two sites with the greatest change in blocking activity in the α6/α3 single-point mutations) was prepared in the hope that the effect of the combined action of multiple residues in α6/α3 on TxIB blocking activity could be investigated to some extent. The combined mutant h[α6_V32L, K61R_/α3]β4 showed a significant decrease in TxIB blocking activity, with an IC_50_ value of 4.3 μM, which was 8.1-fold higher compared to that of the WT hα6/α3β4 (Table 6). Oocytes expressing the h[α6_V32L, K61R_/α3]β4 mutant showed an inhibition of the current response to 100 μM ACh following 10 μM TxIB incubation that was 31.95 ± 8.05% (n = 7) (Figure 7A). The blocking activity of TxIB acting on h[α6_V32L, K61R_/α3]β4 and hα6/α3β4_L107V, V115I_ showed that the combined mutation of multiple residues in a single subunit led to some loss of blocking activity with TxIB but not a substantial decrease in activity. Previous studies have shown that nAChRs’ ligand-binding sites mainly present at the interface formed by the α and β subunits; therefore, considering that α and β, together, affect the interaction with the ligand, h[α6_V32L, K61R_/α3]β4_L107V, V115I_ was prepared to investigate the molecular mechanisms underlying the apparently different activities of TxIB in human and rat α6/α3β4 [24]. The results showed a blocking effect of TxIB on h[α6_V32L, K61R_/α3]β4_L107V, V115I_ with an IC_50_ value of 22.5 μM, a 42-fold decrease in blocking potency compared to the WT hα6/α3β4 nAChR. Oocytes expressing the h[α6_V32L, K61R_/α3]β4_L107V, V115I_ mutant showed 60.16 ± 8.66% (n = 8) inhibition of the current response to 100 μM ACh after 5 min of incubation in the presence of 10 μM TxIB, and under the same conditions, the value of the WT hα6/α3β4 nAChR was 10.99 ± 5.9% (n = 16) (Figure 3B). Multiresidue mutations in α6/α3β4 did not affect binding to TxIB, and the currents blocked by TxIB were fully restored within 1 min (Figure 7A–C). Based on the above results, a single subunit does not determine the difference in activity of TxIB acting on human and rat α6/α3β4 nAChRs; rather, this is the result of the combined action of two subunits. The interaction of 32-Val and 61-Lys, located in the α6/α3 subunit, and that of 107-Leu and 115-Val, in the β4 subunit, together, determine the species specificity of TxIB in human and rat α6/α3β4 nAChRs, constituting the key site for binding to TxIB.

## 3. Discussion

Many α-conotoxins have differential activity in human murine nAChRs, such as PeIA, PnIA, Vc1.1, LvIC, and, as used in our report, TxIB, where the activity of TxIB varies by more than 30-fold, meaning that this is a problem that cannot easily be ignored in the study of conotoxins [1,16,32]. The α-conotoxins are the first and most widely studied members of the conotoxin peptide superfamily. They have been used to develop a variety of pharmacological tools and occupy a position in the development of new drug leads. The clinical activity observed in the development of Vc1.1 in previous reports was much lower than that observed in the rat analgesic model, which shows the significance of studying human–rodent species differences in nAChRs, especially in rodent experimental models that are widely used in drug development and basic pharmacological research [1,16,33]. The α6/α3β4 nAChR function has not been examined and is, to some extent, affected by species differences in the receptor. The human–rat species differences in α6/α3β4 nAChR provide a pharmacological basis for drug development targeting this receptor, which may lead to successful development [23,34].

**Figure 8:**
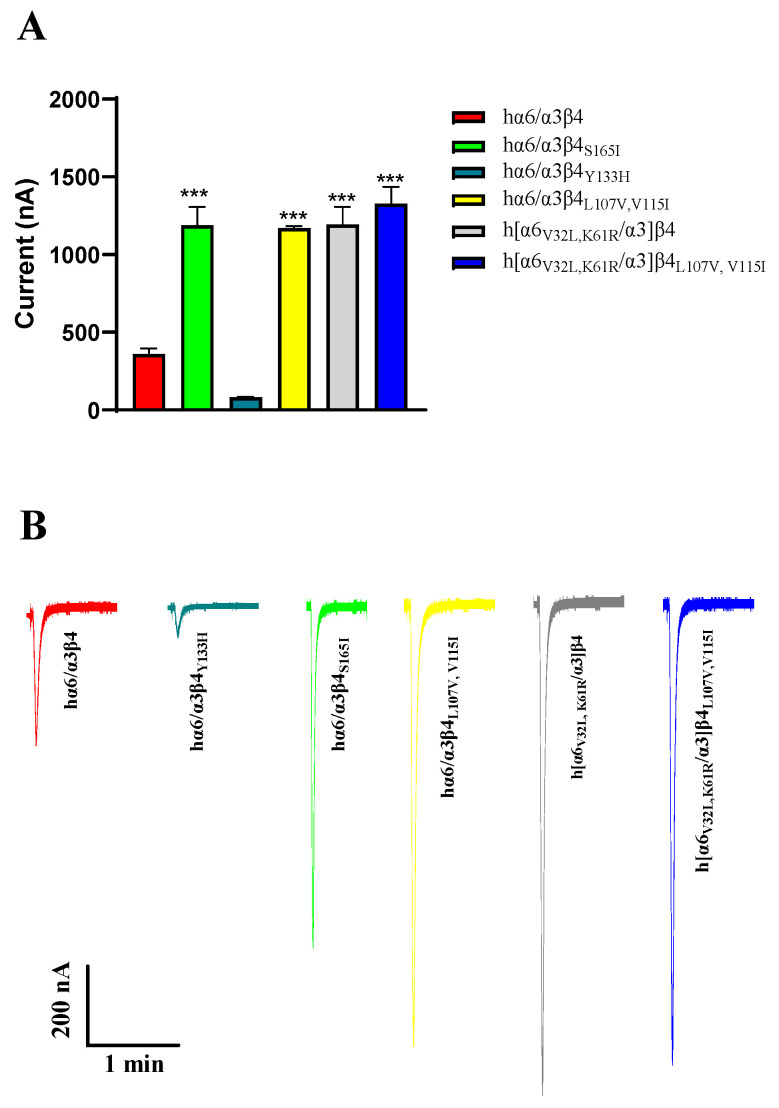
Analysis of the current amplitude of hα6/α3β4 nAChR and its mutants (**A**,**B**). Current magnitude significance analysis. Injection of the same amount of RNA, with the current amplitude at the same expression time. *** represents a significant difference at *p* < 0.001 (**A**). Representative ACh-evoked currents obtained in the presence of 100 μM ACh for hα6/α3β4 and its mutants (**B**).

Regarding current amplitude, the N52S mutation does not form a functional receptor; it has almost no current under agonist stimulation. In our experiments, we also observed that the current amplitude of hα6/α3β4_Y133H_ is decreased, and the current amplitude of hα6/α3β4_S165I_ is increased significantly compared to the WT hα6/α3β4. Similar phenomena were also observed in hα6/α3β4_L107V, V115I_, h[α6_V32L, K61R_/α3]β4, and h[α6_V32L, K61R_/α3]β4_L107V,V115I_, which all showed a significant increase in current amplitude (Figure 8A,B). In contrast, the current amplitude of the remaining hα6/α3β4 nAChR mutants did not change noticeably compared to the WT hα6/α3β4 under the same conditions. In the case of the above phenomena that we observed, we believe that the mutation may have caused a change in the receptor function, possibly because the structure of the receptor pore was changed after the mutation, making it easy for internal and external ion exchange to occur. Additionally, we believe that this situation may be due to a change in the number of functional receptors formed by the mutated receptors on the cell membrane surface.

α6/α3β4 nAChR has been widely recognized in previous studies as having key effects on the amino acid residues in loops D, E, and F in the β4 subunit that build the ligand-binding pocket. Hone, studying α6β4 nAChR species differences according to the mixed expression of human and rat α6 and β4 subunits to determine the main reason for the contribution of the β4 subunit to these species differences, found that the mutation of a nonconserved amino acid at position 116 produced a significant decrease in activity (nearly 10-fold) [11,23,35]. Our subsequent assay of the activity of the L107V and V115I comutations showed that the interaction of Leu at position 107 with Val at position 115 contributed to the difference in activity, with a 4.9-fold decrease in activity (Table 6). Unlike our species difference study using TxIB as a blocking drug, the activity of Leu-Gln at position 116 decreased by only 1.7-fold, which may have been determined by the structural differences between TxIB and PeIA. The mutational activity assay of the remaining amino acid difference sites located in the ligand-binding pocket in β4 revealed that the IC_50_ varied within a small range, and we could not identify sites in the ligand-binding region that were decisive for the species differences. We thus examined the nine remaining differential loci in β4 using the same method to investigate whether other differential loci outside loops D, E, and F affect the activity difference through indirect effects, as shown in Table 1. With such results, we could not conventionally assume that the β4 subunit is more critical than the α6 subunit. We used the same method to search for key amino acid sites in the α6 subunit, resulting in a 3.4-fold decrease in activity after the mutation of Lys into Arg at position 61, a 4.1-fold reduction in activity after the modification of Val-Leu at position 32, and an 8.1-fold decrease in activity after combined mutation, indicating that the α6/α3 subunit plays a more important role in the species differences. Meanwhile, in previous studies, there was no indication that Val-32 may change the ligand affinity by indirectly affecting the subunit tertiary structure, but on the other hand, it was found that this key site located outside the ligand-binding domain may also change the surface of the ligand-binding domain by influencing the interactions between subunits [31]. It was also found that the interaction of the α6/α3 subunit with the key site in the β4 subunit has a significant effect on the activity of the receptor, with a 42-fold decrease in activity, and plays a decisive role in shaping the potency difference between human and rat α6/α3β4 nAChRs.

The use of α6/α3 instead of the wild-type α6* subunit nAChRs, based on previous studies, would involve a slight degree of limitation because of the overall structural variation in nAChRs and poor selectivity resulting from the many ligands of α3* versus α6* nAChRs [14,15]. In order to eliminate the potential effects mentioned above, studies are currently being conducted to promote the expression of native α6* nAChRs through molecular chaperones, including β-anchoring and regulatory protein (BARP), lysosomal-associated membrane protein 5 (LAMP5), and SULT2B1, which complement the nAChR chaperone NACHO to reconstitute the α6β2β3 and α6β4 channel function [36,37].

α6β4* nAChRs have emerged as targets for chronic pain drug development through direct action and cross-inhibition with P2X2/3 receptors [38]. Little information is available on the interaction of ligands with this subtype at the molecular level. The study of selective ligands for α6β4* nAChRs may be critical for avoiding off-target effects due to interactions with their closely related subtypes, particularly α6β2 and α3β4 nAChRs [29]. Our report comprehensively identifies the key amino acid residues associated with differences in α6/α3β4 nAChR species properties, especially the α6 subunit. It is the first to report on the key amino acid sites of the α6 subunit in regard to α6/α3β4 nAChR species differences and to comprehensively mutate all the amino acid difference sites in the α6 and β4 subunits, providing a molecular basis for the elimination of off-target effects in drug development targeting α6/α3β4 nAChR. In summary, we demonstrated that Val-32 and Lys-61 in the α6 subunit and Leu-107 and Val-115 in the β4 subunit are essential amino acid residues for interactions with conotoxin. The information obtained in this study may eventually guide the design of ligands targeting α6/α3β4 nAChRs for the treatment of neuropathic pain, providing crucial information [13,39].

## 4. Materials and Methods

### 4.1. Materials

The plasmid extraction kit used in the experiments, restriction enzymes, and the competent cells, DH5α, used in the cell transformation, were purchased from TaKaRa (Dalian, China), while the cRNA mMESSAGE mMACHINE in vitro transcription kit and the RNA MEGA Clear kit were purchased from Thermo Fisher Scientific (Pittsburgh, PA, USA). Acetylcholine, atropine, BSA, and collagenase were purchased from Sigma-Aldrich (St. Louis, MO, USA). Trifluoroacetic acid (TFA) was purchased from Tedia (Fairfield, OH, USA). All amino acids and chemical reagents used for peptide synthesis were analytically pure. Reversed-phase HPLC (RP-HPLC) analytical Vydac C18 (5 μm, 4.6 mm × 250 mm) and preparative C18 Vydac (10 μm, 22 mm × 250 mm) columns were obtained from Grace Vydac (Hesperia, CA, USA).

Plasmids containing human and rat α6/α3 and β4 nAChR subunit genes were obtained from the University of Utah, USA. α6/α3 indicates chimeric (formed by splicing the N-terminal extracellular domain of the α6 subunit with the α3 subunit) receptors, as with wild-type α6* receptors, it is challenging to create a functional expression in *X. Laevis* oocytes [3].

The female *Xenopus laevis* used for the experiments were obtained from the Kunming Institute of Zoology (Kunming, China) and were fed twice a week and kept at 17 °C for over 6 months. All animal-related operations followed the Animal Ethics Committee of Guangxi University guidelines. Mature female *X. Laevis* frogs were anesthetized on ice, and the oocytes were prepared as previously described [40].

### 4.2. Peptide Synthesis

The TxIB linear peptides were synthesized by GL Biochem Ltd., (Shanghai, China). The TxIB linear peptides were oxidized in two steps as previously described [41]. The disulfide bond was synthesized using a two-step oxidation method in 20 nmol/L potassium ferricyanide, 0.1 mol/L Tris, and pH = 7.5 solution. The monocyclic peptide formed in the first step of TxIB oxidation was separated and purified via reversed-phase high-performance liquid chromatography (RP-HPLC) for 45 min. The collected monocyclic peptide was oxidized in the second step, and the monocyclic peptide was added dropwise to a solution containing 1 mmol/L iodine and stirred under nitrogen protection for 10 min. The iodine was oxidized to form the 2nd disulfide bond. HPLC was conducted to analyze the samples, and the main peaks were collected and identified using mass spectrometry. The products were purified via HPLC on a reversed-phase C18 Vydac column using a linear gradient of 5–95% buffer B (0.05% TFA and 90% acetonitrile in ddH_2_O) over 50 min. Buffer A was 0.075% TFA in ddH_2_O. The purity of the peptide was determined via UV monitoring at an absorbance of 214 nm during HPLC (≥95% purity). Mass spectrometry was utilized to confirm the molecular mass of the TxIB.

### 4.3. Site-Directed Mutagenesis of α6/α3 and β4 Subunits

Rat and human α6/α3 and β4 subunit N-terminal amino acid sequences were compared and numbered using CLC viewer 6 (CLC bio, Aarhus, Denmark). Mutants were mutated using a PCR-mediated single-point mutation method. The primers were designed using primer premier 5.0 software (Premier Biosoft International, Palp Alto, CA, USA) and synthesized by Biotech Biological Engineering Co. (Shanghai, China). Primers containing the desired point mutation flanked by 12–22 bases on either side were synthesized by Sangon Biotech Co. Ltd. (Shanghai, China). The PCR conditions were as follows: 95 °C denaturation for 2 min, followed by 20 cycles of 95 °C for 20 s, 60 °C for 10 s, and 68 °C for 3 min, and a final extension at 68 °C for 5 min. The *Dpn* I digestion reaction was then performed to remove the template cDNA, subsequently transformed using the Dh5α receptor cells, coated in agar medium containing ampicillin, and cultured overnight at 37 °C. Five single colonies were selected from each medium, verified via sequencing by Biotech Bioengineering, and expanded to extract the cDNA containing mutant sites using a plasmid extraction kit.

### 4.4. cRNA Synthesis and Injection

The hα6/α3, hβ4, and rβ4 subunits were linearized using *Nhe* I. The rα6/α3 subunits were prepared using *Sal* I for linearized cDNA. cRNA was prepared from the linearized cDNA using the mMESSAGE mMACHINE transcription kit and purified using the MEGAclear kit. In all subsequent experiments, oocytes were injected with a 1:1 ratio of cRNA, with single-subunit injections greater than 50 ng per oocyte to ensure correct receptor expression. The injected oocytes were cultured in a constant-temperature incubator, and electrophysiological assays were performed 3–7 days after injection. The oocytes’ culture solution was ND96 (96 mM NaCl, 2 mM KCl, 1.8 mM CaCl_2_, 1 mM MgCl_2_, and 5 mM HEPES, pH 7.1–7.5).

### 4.5. Electrophysiological Assay

Oocytes injected with the corresponding cRNA were assayed at room temperature, fixed in 50 μL of ND96-filled chambers, and perfused continuously at 3 mL/min. To apply ACh pulses to the oocytes, the perfusate was replaced with ND96 fluid containing 100 μM ACh using a dispensing valve at a 3 mL/min perfusion rate for 2 s. This procedure was automatically performed at intervals of 60 s. The response to ACh alone, before treatment with conotoxin, was taken as the control response. The current response to agonist ACh application was measured using a two-electrode voltage clamp amplifier 1050B at a holding voltage of −70 mV. Micropipettes were filled with 3 M KCl and had resistances of 0.5–2 MΩ. The agonist-induced current responses were recorded and analyzed using pClamp11.2 software. The blockade of TxIB was determined by comparing the ACh-induced current response after 5 min of incubation with conotoxins to the average of three ACh-induced peak current responses preceding conotoxin incubation.

### 4.6. Data Analysis

The current magnitude of oocytes under the effect of different concentrations of conotoxin was determined. The response rate after drug action was calculated based on the current produced by the oocytes at 100 μM acetylcholine before drug administration. The response rates at the given drug concentrations for groups 6–9 were substituted into the dose–response curves. α-CTxs were applied only after the ACh response-to-response variation was 10%. The variance in the responses is provided as the mean ± SEM and shown with error bars. α-CTx data were replicated for different batches of oocytes to ensure the reproducibility of the data. To calculate the IC_50_ values, the normalized data were analyzed via nonlinear fitting, and a four-parameter logistic equation was performed using GraphPad Prism 8.0. Significance was determined at the 95% level (*p* < 0.05). The concentration–response curves for the activation of the nAChRs were determined at increasing ACh concentrations to assess the magnitude of the induced currents. The response rate at each acetylcholine concentration was calculated based on the maximum induced currents, and finally, the four-parameter logistic equation was performed. Acetylcholine curve data were also obtained in three batches to ensure reproducibility.

## Figures and Tables

**Table 1:** EC_50_ values of ACh for hα6/α3β4 and its β4 mutants.

nAChRs	EC_50_ (95% CI) ^a^ (μM)	Hill Slope	Ratio ^b^
hα6/α3β4	138 (121–158)	1.4 (1.2–1.7)	1
hα6/α3β4_K31R_	129 (111–150)	1.3 (1.1–1.6)	0.9
hα6/α3β4_Q33E_	173 (151–199)	1.2 (1.1–1.5)	1.3
hα6/α3β4_A37S_	94 (80.6–111)	1.1 (1.0–1.4)	0.7
hα6/α3β4_V53I_	95 (86–108)	1.6 (1.2–2.3)	0.7
hα6/α3β4_T65A_	157 (137–183)	1.3 (1.1–1.6)	1.1
hα6/α3β4_R70C_	126 (111–141)	1.2 (1.1–1.5)	0.9
hα6/α3β4_I84V_	135 (119–157)	1.1 (1.0–1.4)	0.9
hα6/α3β4_L107V_	65 (55–75)	1.9 (1.1–3.1)	0.4
hα6/α3β4_V115I_	112 (97–149)	1.4 (1.0–2.2)	0.8
hα6/α3β4_L116Q_	161 (140–184)	1.4 (1.2–1.7)	1.1
hα6/α3β4_Y133H_	195 (160–237)	1.2 (0.9–1.4)	1.4
hα6/α3β4_M160K_	96 (69–131)	0.9 (0.7–1.3)	0.7
hα6/α3β4_T161S_	163 (139–192)	1.1 (0.9–1.4)	1.2
hα6/α3β4_S165I_	51 (41–58)	2.9 (1.8–4.9)	0.4

^a^ The 95% confidence intervals for EC_50_ values; ^b^ change in acetylcholine (ACh) EC_50_ values relative to hα6/α3β4. Hill slopes obtained from ACh concentration–response curves for WT and mutant hα6/α3β4. All data represent the mean ± SEM, n = 6–9.

**Table 2:** IC_50_ values of TxIB for hα6/α3β4 and its β4 subunit mutants.

nAChRs	IC_50_ (95% CI) ^a^ (nM)	Hill Slope	Ratio ^b^
hα6/α3β4	537 (492–588)	0.8 (0.8–0.9)	1
hα6/α3β4_K31R_	1173 (994–1394)	0.8 (0.7–0.9)	2.2
hα6/α3β4_Q33E_	446 (359–553)	0.9 (0.7–1.0)	0.8
hα6/α3β4_A37S_	559 (507–616)	0.9 (0.8–1.0)	1.1
hα6/α3β4_V53I_	589 (531–655)	0.8 (0.7–0.9)	1.1
hα6/α3β4_T65A_	551 (491–619)	0.9 (0.8–1.0)	1
hα6/α3β4_R70C_	503 (430–590)	0.8 (0.7–1.0)	0.9
hα6/α3β4_I84V_	767 (631–946)	0.8 (0.8–0.9)	1.4
hα6/α3β4_L107V_	1760 (620–1914)	0.8 (0.7–0.9)	3.3
hα6/α3β4_V115I_	1525 (1388–1742)	0.8 (0.7–0.9)	2.8
hα6/α3β4_L116Q_	910 (794–1048)	0.8 (0.7–0.9)	1.7
hα6/α3β4_Y133H_	681 (542–864)	0.8 (0.7–1.0)	1.3
hα6/α3β4_M160K_	825 (680–1017)	0.9 (0.7–1.1)	1.5
hα6/α3β4_T161S_	977 (775–1243)	0.8 (0.6–0.9)	1.8
hα6/α3β4_S165I_	547 (464–645)	0.7 (0.6–0.9)	1

^a^ The 95% confidence intervals for IC_50_ values; ^b^ change in TxIB IC_50_ values relative to hα6/α3β4. Hill slopes obtained from TxIB concentration–response curves for WT and mutant hα6/α3β4. All data represent the mean ± SEM, n = 6–12.

**Table 3:** EC_50_ values of ACh for hα6/α3β4 and its α6/α3 mutants.

nAChRs	EC_50_ (95% CI) ^a^ (μM)	Hill Slope	Ratio ^b^
hα6/α3β4	138 (121–158)	1.4 (1.2–1.7)	1
h[α6_R3Q_/α3]β4	137 (117–159)	1.4 (1.2–1.8)	1
h[α6_K7T_/α3]β4	154 (127–18)	1.5 (1.1–1.9)	1.1
h[α6_S10A_/α3]β4	175 (142–217)	1.6 (1.2–2.3)	1.3
h[α6_Q14R_/α3]β4	216 (191–243)	1.4 (1.2–1.6)	1.6
h[α6_V32L_/α3]β4	167 (146–192)	1.5 (1.3–1.9)	1.2
h[α6_I56V_/α3]β4	153 (136–173)	1.3 (1.1–1.5)	1.1
h[α6_N58K_/α3]β4	176 (156–199)	1.4 (1.2–1.7)	1.3
h[α6_K61R_/α3]β4	202 (178–229)	1.4 (1.2–1.7)	1.5
h[α6_R63C_/α3]β4	150 (131–173)	1.5 (1.2–1.8)	1.1
h[α6_M67T_/α3]β4	122 (91–166)	1.4 (0.9–2.2)	0.9
h[α6_K81N_/α3]β4	171 (154–190)	1.4 (1.3–1.7)	1.2
h[α6_N110D_/α3]β4	182 (146–227)	1.2 (0.9–1.6)	1.3
h[α6_M112V_/α3]β4	109 (94–128)	1.4 (1.1–1.8)	0.8
h[α6_I176V_/α3]β4	125 (130–139)	1.7 (1.4–2.1)	0.9

^a^ The 95% confidence intervals for the EC_50_ values; ^b^ change in acetylcholine (ACh) EC_50_ values relative to hα6/α3β4. Hill slopes obtained from ACh concentration–response curves for the WT and mutant hα6/α3β4. All data represent the mean ± SEM, n = 6–9.

**Table 4:** IC_50_ values of TxIB for hα6/α3β4 and its α6/α3 subunit mutants.

nAChRs	IC_50_ (95% CI) ^a^ (nM)	Hill Slope	Ratio ^b^
hα6/α3β4	537 (492–588)	0.8 (0.8–0.9)	1
h[α6_R3Q_/α3]β4	486 (434–545)	0.9 (0.8–1.1)	0.9
h[α6_K7T_/α3]β4	732 (615–876)	0.8 (0.6–0.9)	1.4
h[α6_S10A_/α3]β4	624 (529–737)	0.8 (0.7–0.9)	1.2
h[α6_Q14R_/α3]β4	514 (421–629)	0.9 (0.7–1.2)	1
h[α6_V32L_/α3]β4	2283 (1985–2631)	0.7 (0.7–0.8)	4.2
h[α6_I56V_/α3]β4	489 (427–559)	1.0 (0.9–1.2)	0.9
h[α6_N58K_/α3]β4	471 (409–541)	0.9 (0.8–1.1)	0.9
h[α6_K61R_/α3]β4	1833 (1609–2097)	0.8 (0.7–0.9)	3.4
h[α6_R63C_/α3]β4	512 (439–599)	0.9 (0. 8–1.1)	0.9
h[α6_M67T_/α3]β4	567 (483–668)	0.9 (0.7–1.0)	1.1
h[α6_K81N_/α3]β4	634 (561–718)	0.8 (0.7–0.9)	1.2
h[α6_N110D_/α3]β4	884 (808–970)	0.9 (0.8–1.0)	1.6
h[α6_M112V_/α3]β4	337 (293–388)	0.8 (0.7–0.9)	0.6
h[α6_I176V_/α3]β4	451 (388–523)	0.8 (0.7–0.9)	0.8

^a^ The 95% confidence intervals for IC_50_ values; ^b^ change in TxIB IC_50_ values relative to hα6/α3β4. Hill slopes obtained from TxIB concentration–response curves on WT and mutant hα6/α3β4. All data represent the mean ± SEM, n = 6–16.

**Table 5:** EC_50_ values of ACh for hα6/α3β4 and its combined mutants.

nAChRs	EC_50_ (95% CI) ^a^ (μM)	Hill Slope	Ratio ^b^	n
hα6/α3β4	138 (121–158)	1.4 (1.2–1.7)	1	9
hα6/α3β4_L107V, V115I_	94 (79–112)	1.5 (1.1–2.1)	0.7	10
h[α6_V32L, K61R_/α3]β4	246 (227–267)	1.2 (1.1–1.3)	1.8	7
h[α6_V32L, K61R_/α3]β4_L107V,V115I_	133 (115–154)	1.2 (1.0–1.5)	0.9	7

^a^ The 95% confidence intervals for the EC_50_ values; ^b^ change in acetylcholine (ACh) EC_50_ values relative to hα6/α3β4. Hill slopes obtained from ACh concentration–response curves for the WT and mutant hα6/α3β4. All data represent the mean ± SEM, n = 6–9.

**Table 6:** IC_50_ values of TxIB for hα6/α3β4 and its combined mutants.

nAChRs	IC_50_ (95% CI) ^a^ (nM)	Hill Slope	Ratio ^b^
hα6/α3β4	537 (492–588)	0.9 (0.8–0.9)	1
hα6/α3β4_L107V, V115I_	2608 (2386–2854)	0.8 (0.8–0.9)	4.9
h[α6_V32L, K61R_/α3]β4	4342 (3633–5217)	0.8 (0.7–1)	8.1
h[α6_V32L, K61R_/α3]β4_L107V,V115I_	22,550 (17,860–29,170)	0.8 (0.6–0.9)	42

^a^ The 95% confidence intervals for the IC_50_ values; ^b^ change in TxIB IC_50_ values relative to hα6/α3β4. Hill slopes obtained from TxIB concentration–response curves for the WT and mutant hα6/α3β4. All data represent the mean ± SEM, n = 6–9.

## Data Availability

All data generated or analyzed during this study are included in this published article.

## Abbreviations

ACh, acetylcholine chloride; nAChR, nicotinic acetylcholine receptor; BSA, bovine serum albumin; ESI-MS, electrospray–ionization mass spectroscopy; h, human; RP-HPLC, reversed phase high-performance liquid chromatography; HPLC, high-performance liquid chromatography; r, rat; TFA, trifluoroacetic acid; NMR, nuclear magnetic resonance.

## References

[1] Hone A.J., Talley T.T., Bobango J., Melo C.H., Hararah F., Gajewiak J.B., Christensen S.B., Harvey P.J., Craik D.J., McIntosh J.M. (2018). Molecular determinants of α-conotoxin potency for inhibition of human and rat alpha 6 beta 4 nicotinic acetylcholine receptors. J. Biol. Chem..

[2] Sine S.M., Engel A.G. (2006). Recent advances in Cys-loop receptor structure and function. Nature.

[3] Cox B.C., Marritt A.M., Perry D.C., Kellar K.J. (2008). Transport of multiple nicotinic acetylcholine receptors in the rat optic nerve: High densities of receptors containing alpha6 and beta3 subunits. J. Neurochem..

[4] Azam L., Yoshikami D., McIntosh J.M. (2008). Amino acid residues that confer high selectivity of the alpha6 nicotinic acetylcholine receptor subunit to alpha-conotoxin MII[S4A,E11A,L15A]. J. Biol. Chem..

[5] Papke R.L., Dwoskin L.P., Crooks P.A., Zheng G., Zhang Z., McIntosh J.M., Stokes C. (2008). Extending the analysis of nicotinic receptor antagonists with the study of alpha6 nicotinic receptor subunit chimeras. Neuropharmacology.

[6] Pons S., Fattore L., Cossu G., Tolu S., Porcu E., McIntosh J.M., Changeux J.P., Maskos U., Fratta W. (2008). Crucial role of alpha4 and alpha6 nicotinic acetylcholine receptor subunits from ventral tegmental area in systemic nicotine self-administration. J. Neurosci..

[7] Jackson K.J., McIntosh J.M., Brunzell D.H., Sanjakdar S.S., Damaj M.I. (2009). The role of alpha6-containing nicotinic acetylcholine receptors in nicotine reward and withdrawal. J. Pharmacol. Exp. Ther..

[8] You S., Li X.D., Xiong J., Zhu X.Y., Zhangsun D.T., Zhu X.P., Luo S.L. (2019). alpha-Conotoxin TxIB: A Uniquely Selective Ligand for alpha 6/alpha 3 beta 2 beta 3 Nicotinic Acetylcholine Receptor Attenuates Nicotine-Induced Conditioned Place Preference in Mice. Mar. Drugs.

[9] Yu J.P., Zhu X.P., Yang Y., Luo S.L., Zhangsun D.T. (2018). Expression in Escherichia coli of fusion protein comprising -conotoxin TxIB and preservation of selectivity to nicotinic acetylcholine receptors in the purified product. Chem. Biol. Drug Des..

[10] Quik M., Bordia T., O’Leary K. (2007). Nicotinic receptors as CNS targets for Parkinson’s disease. Biochem. Pharmacol..

[11] Hone A.J., McIntosh J.M. (2018). Nicotinic acetylcholine receptors in neuropathic and inflammatory pain. FEBS Lett..

[12] Hone A.J., Meyer E.L., McIntyre M., McIntosh J.M. (2012). Nicotinic acetylcholine receptors in dorsal root ganglion neurons include the alpha6beta4* subtype. FASEB J..

[13] Marucci G., Dal Ben D., Buccioni M., Marti Navia A., Spinaci A., Volpini R., Lambertucci C. (2019). Update on novel purinergic P2X3 and P2X2/3 receptor antagonists and their potential therapeutic applications. Expert Opin. Ther. Patents.

[14] Kuryatov A., Olale F., Cooper J., Choi C., Lindstrom J. (2000). Human α6 AChR subtypes: Subunit composition, assembly, and pharmacological responses. Neuropharmacology.

[15] Cheryl D., Baldomero M.O., James E.G., Sarah T.S., Maren W., Alexander K., Doju Y., Jon M.L., McIntosh J.M. (2003). α-Conotoxin PIA Is Selective for α6 Subunit-Containing Nicotinic Acetylcholine Receptors. J. Neurosci..

[16] Satkunanathan N., Livett B., Gayler K., Sandall D., Down J., Khalil Z. (2005). Alpha-conotoxin Vc1.1 alleviates neuropathic pain and accelerates functional recovery of injured neurones. Brain Res..

[17] Gao F., Chen D., Ma X., Sudweeks S., Yorgason J.T., Gao M., Turner D., Eaton J.B., McIntosh J.M., Lukas R.J. (2019). Alpha6-containing nicotinic acetylcholine receptor is a highly sensitive target of alcohol. Neuropharmacology.

[18] Kamens H.M., Hoft N.R., Cox R.J., Miyamoto J.H., Ehringer M.A. (2012). The alpha6 nicotinic acetylcholine receptor subunit influences ethanol-induced sedation. Alcohol.

[19] Zhang B.J., Ren M.M., Xiong Y., Li H.N., Wu Y., Fu Y., Zhangsun D.T., Dong S., Luo S.L. (2021). Cysteine [2,4] Disulfide Bond as a New Modifiable Site of alpha-Conotoxin TxIB. Mar. Drugs.

[20] Luo S., Zhangsun D., Wu Y., Zhu X., Hu Y., McIntyre M., Christensen S., Akcan M., Craik D.J., McIntosh J.M. (2013). Characterization of a novel alpha-conotoxin from conus textile that selectively targets alpha6/alpha3beta2beta3 nicotinic acetylcholine receptors. J. Biol. Chem..

[21] Li X., Wang S., Zhu X., Zhangsun D., Wu Y., Luo S. (2020). Effects of Cyclization on Activity and Stability of alpha-Conotoxin TxIB. Mar. Drugs.

[22] Wittenberg R.E., Wolfman S.L., De Biasi M., Dani J.A. (2020). Nicotinic acetylcholine receptors and nicotine addiction: A brief introduction. Neuropharmacology.

[23] Hone A.J., Kaas Q., Kearns I., Hararah F., Gajewiak J., Christensen S., Craik D.J., McIntosh J.M. (2021). Computational and Functional Mapping of Human and Rat alpha6beta4 Nicotinic Acetylcholine Receptors Reveals Species-Specific Ligand-Binding Motifs. J. Med. Chem..

[24] Gharpure A., Teng J., Zhuang Y., Noviello C.M., Walsh R.M., Cabuco R., Howard R.J., Zaveri N.T., Lindahl E., Hibbs R.E. (2019). Agonist Selectivity and Ion Permeation in the alpha3beta4 Ganglionic Nicotinic Receptor. Neuron.

[25] Dash B., Bhakta M., Chang Y.C., Lukas R.J. (2011). Identification of N-terminal Extracellular Domain Determinants in Nicotinic Acetylcholine Receptor (nAChR) alpha 6 Subunits That Influence Effects of Wild-type or Mutant beta 3 Subunits on Function of alpha 6 beta 2*-or alpha 6 beta 4*-nAChR. J. Biol. Chem..

[26] Noviello C.M., Gharpure A., Mukhtasimova N., Cabuco R., Baxter L., Borek D., Sine S.M., Hibbs R.E. (2021). Structure and gating mechanism of the alpha 7 nicotinic acetylcholine receptor. Cell.

[27] Hone A.J., Fisher F., Christensen S., Gajewiak J., Larkin D., Whiteaker P., McIntosh J.M. (2019). PeIA-5466: A Novel Peptide Antagonist Containing Non-natural Amino Acids That Selectively Targets alpha3beta2 Nicotinic Acetylcholine Receptors. J. Med. Chem..

[28] Hone A.J., Scadden M., Gajewiak J., Christensen S., Lindstrom J., McIntosh J.M. (2012). alpha-Conotoxin PeIA[S9H,V10A,E14N] potently and selectively blocks alpha6beta2beta3 versus alpha6beta4 nicotinic acetylcholine receptors. Mol. Pharmacol..

[29] Zhu X.P., Wang S., Kaas Q., Yu J.P., Wu Y., Harvey P.J., Zhangsun D., Craik D.J., Luo S.L. (2023). Discovery, Characterization, and Engineering of LvIC, an alpha 4/4-Conotoxin That Selectively Blocks Rat alpha 6/alpha 3 beta 4 Nicotinic Acetylcholine Receptors. J. Med. Chem..

[30] Exley R., Maubourguet N., David V., Eddine R., Evrard A., Pons S., Marti F., Threlfell S., Cazala P., McIntosh J.M. (2011). Distinct contributions of nicotinic acetylcholine receptor subunit alpha4 and subunit alpha6 to the reinforcing effects of nicotine. Proc. Natl. Acad. Sci. USA.

[31] Luo S.L., Zhangsun D.T., Schroeder C.I., Zhu X.P., Hu Y.Y., Wu Y., Weltzin M.M., Eberhard S., Kaas Q., Craik D.J. (2014). A novel alpha 4/7-conotoxin LvIA from Conus lividus that selectively blocks alpha 3 beta 2 vs. alpha 6/alpha 3 beta 2 beta 3 nicotinic acetylcholine receptors. FASEB J..

[32] Kasheverov I.E., Chugunov A.O., Kudryavtsev D.S., Ivanov I.A., Zhmak M.N., Shelukhina I.V., Spirova E.N., Tabakmakher V.M., Zelepuga E.A., Efremov R.G. (2016). High-Affinity alpha-Conotoxin PnIA Analogs Designed on the Basis of the Protein Surface Topography Method. Sci. Rep..

[33] Mohammadi S.A., Christie M.J. (2015). Conotoxin Interactions with alpha9alpha10-nAChRs: Is the alpha9alpha10-Nicotinic Acetylcholine Receptor an Important Therapeutic Target for Pain Management?. Toxins.

[34] Sandager-Nielsen K., Ahring P.K., Klein J., van Hout M., Thaneshwaran S., Dos Santos A.B., Jacobsen T.A., Amrutkar D.V., Peters D., Jensen A.A. (2020). Characterization of AN317, a novel selective agonist of alpha6beta2-containing nicotinic acetylcholine receptors. Biochem. Pharmacol..

[35] Hogg R.C., Raggenbass M., Bertrand D. (2003). Nicotinic acetylcholine receptors: From structure to brain function. Rev. Physiol. Biochem. Pharmacol..

[36] Gu S., Matta J.A., Davini W.B., Dawe G.B., Lord B., Bredt D.S. (2019). alpha6-Containing Nicotinic Acetylcholine Receptor Reconstitution Involves Mechanistically Distinct Accessory Components. Cell Rep..

[37] Knowland D., Gu S., Eckert W.A., Dawe G.B., Matta J.A., Limberis J., Wickenden A.D., Bhattacharya A., Bredt D.S. (2020). Functional alpha6beta4 acetylcholine receptor expression enables pharmacological testing of nicotinic agonists with analgesic properties. J. Clin. Investig..

[38] Bernier L.P., Ase A.R., Seguela P. (2018). P2X receptor channels in chronic pain pathways. Br. J. Pharmacol..

[39] Unwin N. (2005). Refined structure of the nicotinic acetylcholine receptor at 4A resolution. J. Mol. Biol..

[40] Almouzni G., Wolffe A.P. (1993). Nuclear assembly, structure, and function: The use of Xenopus in vitro systems. Exp. Cell Res..

[41] Wu X.S., Wu Y., Zhu F.R., Yang Q.Y., Wu Q.Q., Zhangsun D.T., Luo S.L. (2013). Optimal Cleavage and Oxidative Folding of alpha-Conotoxin TxIB as a Therapeutic Candidate Peptide. Mar. Drugs.

